# Canalization of gene expression is a major signature of regulatory cold adaptation in temperate *Drosophila melanogaster*

**DOI:** 10.1186/s12864-016-2866-0

**Published:** 2016-08-08

**Authors:** Korbinian von Heckel, Wolfgang Stephan, Stephan Hutter

**Affiliations:** Department of Biology II, University of Munich (LMU), Grosshaderner Str. 2, 82152 Planegg-Martinsried, Germany

**Keywords:** Transcriptomics, Adaptation, Canalization, Cold tolerance

## Abstract

**Background:**

Transcriptome analysis may provide means to investigate the underlying genetic causes of shared and divergent phenotypes in different populations and help to identify potential targets of adaptive evolution. Applying RNA sequencing to whole male *Drosophila melanogaster* from the ancestral tropical African environment and a very recently colonized cold-temperate European environment at both standard laboratory conditions and following a cold shock, we seek to uncover the transcriptional basis of cold adaptation.

**Results:**

In both the ancestral and the derived populations, the predominant characteristic of the cold shock response is the swift and massive upregulation of heat shock proteins and other chaperones. Although we find ~25 % of the genome to be differentially expressed following a cold shock, only relatively few genes (*n* = 16) are up- or down-regulated in a population-specific way. Intriguingly, 14 of these 16 genes show a greater degree of differential expression in the African population. Likewise, there is an excess of genes with particularly strong cold-induced changes in expression in Africa on a genome-wide scale.

**Conclusions:**

The analysis of the transcriptional cold shock response most prominently reveals an upregulation of components of a general stress response, which is conserved over many taxa and triggered by a plethora of stressors. Despite the overall response being fairly similar in both populations, there is a definite excess of genes with a strong cold-induced fold-change in Africa. This is consistent with a detrimental deregulation or an overshooting stress response. Thus, the canalization of European gene expression might be responsible for the increased cold tolerance of European flies.

**Electronic supplementary material:**

The online version of this article (doi:10.1186/s12864-016-2866-0) contains supplementary material, which is available to authorized users.

## Background

Phenotypic variability in nature is constrained. Strikingly uniform phenotypes appear in high frequency in a population despite genetic and environmental variation. The phenomenon that results in this phenotypic robustness has been termed canalization [1]. Its conception is diverse (see [2] for a review) and debated in many aspects, e.g. whether or not it is necessarily promoted by natural selection [3]. Canalization is often invoked with regard to the morphological development of an organism, but likewise applies throughout adult stages (e.g. [4]) and to more subtle phenotypic features like gene expression [5–7]. Here, we report evidence for a case of environmental canalization, namely for a decreased thermosensitivity of gene expression in temperate *Drosophila melanogaster*.

*D. melanogaster*-nowadays a cosmopolitan human commensal-is of afro-tropical origin and has colonized temperate habitats only after the last glaciation event about 15,000 years ago [8, 9]. Adaptation to the colder climate has likely been one of the major steps facilitating this range expansion [10]. When raised under common lab conditions, flies from temperate habitats show an increased cold resistance compared to flies from tropical origin, as usually determined by a shorter chill coma recovery time (CCRT) [11, 12]. Regulatory evolution presumably accounts for a considerable share of the genetic basis of this adaptive phenotypic difference [13–16]. Hence, investigations of the transcriptome, which serves as a link between genotype and phenotype, may provide important insights into the adaptive process and may help to identify particular genes that have undergone regulatory evolution to confer greater cold tolerance.

Genome-wide expression analyses to uncover regulatory differences between ancestral and derived *D. melanogaster* populations have been mostly conducted at standard lab conditions [17, 18]. It is, however, anticipated that a huge share of regulatory differences regarding environmental stressors is not apparent under standard conditions, but only becomes detectable when the respective stress, in this case a cold shock, is applied. Moreover, many transcriptomic studies regarding cold tolerance in *D. melanogaster* have been performed in a single [19, 20] or few and often old lab strains. Differences in cold tolerance between these strains are likely the result of random genetic drift due to relaxed environmental constraints and inbreeding [21] or have been established due to artificial selection [22]. In contrast, we employed natural populations of *D. melanogaster* that have spent considerably less time in the laboratory and that differ phenotypically because they are adapted to vastly different environments.

To be able to observe a good portion of the adaptive change, we sought to employ populations from the climatic extremes of the species range, which also represent ancestral and derived cases with respect to the demographic process of range expansion and global dispersal: The Siavonga population (ZI) from Zambia stems from a truly tropical environment and shows the highest genetic variation that has been observed for any *D. melanogaster* population to date [23]. Thus, this population is potentially close to the source of all extant cosmopolitan *D. melanogaster* populations. The Swedish population from Umea (SU), on the contrary, constitutes a derived case from the northernmost tip of the species range (unpublished results). As expected, CCRT in the Zambian population is consistently and considerably longer than for the Swedish flies. Since CCRT is heavily influenced by plastic changes due to the environment [12], we verified population differences over a range of environmental conditions (Additional file 1: Table S1).

In an effort to uncover the underlying genetic regulatory basis of this phenotypic difference, we performed high-throughput RNA sequencing of mRNA extracted from whole male flies of the two populations at four different experimental conditions: before, during and 15 & 90 min in the recovery phase after a 7 h cold-shock. Whereas the timepoint before the cold shock serves as a baseline control, the 15 min timepoint was chosen, because we anticipated that some of the expression change at this time might be directly related to the process of recovery itself, since this is also the timepoint when the first fast-recovering European flies tend to wake up from their cold-induced coma [24]. The 90 min timepoint, on the other hand, was chosen, because we established in a set of preceding qPCR experiments that many of the genes that are known to strongly respond towards a cold shock peak in expression around this timepoint (see also [25–27]). We performed RNAseq on three Swedish strains with particular short CCRT and on three Zambian strains with particular long CCRT. To broaden the scope, we included one additional fast-recovering European strain from the Netherlands and one additional slow-recovering African strain from Zimbabwe. The latter two have been previously employed in QTL analyses to identify genes affecting cold tolerance [24, 28].

Exploring the transcriptomic data, we determined genes that are differentially expressed due to either experimental condition or due to continental origin, and, most interestingly, genes that respond to the cold shock in a population-specific way, i.e. genes that exhibit a genotype by environment interaction (GEI). To our knowledge, this is the first study in which the genome-wide transcriptional response to a cold shock is measured and compared between a derived cold tolerant European and an ancestral cold sensitive African population of *D. melanogaster*.

Finally, to evaluate the functional implications of different mRNA levels, we artificially knocked down gene expression for several individual genes via ubiquitous RNA interference employing the VDRC RNAi library [29]. We then compared CCRT and the survival rate 24 h after a cold shock in the knockdown and a control following the experimental approach described elsewhere [25]. So far, the use of this methodology has lead to contradictory results. While some observed clear-cut phenotypic effects on cold tolerance due to the knockdown of a specific gene [25], others were largely unable to replicate these findings [30].

## Results

### Phenotypic population differences in cold tolerance

**Fig. 1:**
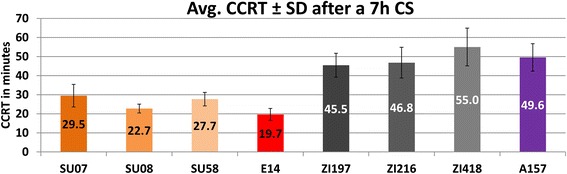
CCRT for the eight focal strains. Chill coma recovery time (CCRT) was determined following a 7 h cold shock in an ice-water bath. Depicted values are averaged over both sexes and a multitude of independent experiments. Strains originate from Umea, Sweden (SU07, SU08, SU58), Leiden, the Netherlands (E14), Siavonga, Zambia (ZI197, ZI216, ZI418), and Lake Kariba, Zimbabwe (A157)

When reared under common lab conditions, the mean CCRT after a 7 h cold shock without any pretreatment, averaged over all strains, both sexes and a multitude of independent experiments is 28.3–29.2 min (95 % CI, *n* = 484; every n represents the average CCRT of ~10 flies) for the Swedish and 41.1–42.8 (95 % CI, *n* = 437) minutes for the Zambian population. This is the condition that was also used in the transcriptome analyses via RNAseq and qRT-PCR. For the eight focal strains of this study the individual CCRT at this condition is shown in Fig. 1. In a different set of experiments we varied environmental conditions by rearing flies at 17 °C and/or by including an acclimation treatment before the cold shock (12 h 6 °C). CCRT is heavily influenced by these environmental alterations, but notably, differences between populations persist (Additional file 1: Table S1).

### Transcriptome overview

To investigate population differences in the transcriptional cold shock response, 5 day old male flies of the four African and the four European strains were subjected to a 7 h cold shock. Total RNA was isolated from whole flies of each strain at four distinct timepoints: before the cold shock (RT), 3.5 h into the cold shock (CS) and 15 & 90 min after flies have been brought back to room temperature (rec15, rec90). After library preparation and sequencing we obtained, in total, over 1.8 billion 51 bp reads from 64 cDNA libraries, which comprise two biological replicates of each strain-and-timepoint-combination. Read quality is generally very high, with a mean Phred score of 35.7. Notably, for the first and last positions the mean Phred score does not drop below 30. Overall, more than 90 % of the reads map to annotated transcripts, just under 4 % map to rRNA, about 1 % to other noncoding RNAs, and a little over 4 % of all reads could not be mapped to the *D. melanogaster* genome. Of the 13,955 annotated protein-coding genes in FlyBase release 5.57 [31], 13,821 have at least one mapped read in at least one library, whereas 12,617 genes have at least one mapped read in every library.

**Fig. 2:**
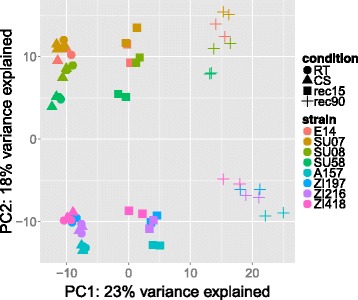
Transcriptome overview: PCA. PCA was calculated using the built-in methods provided by DESeq2 [42] for variance stabilizing transformation of read counts and PCA on the 500 genes with the highest overall expression variance. Note that samples are clearly separated according to continent and condition with the exception of RT and CS samples, which cluster tightly together in both populations such that symbols partly overlap

A principle component analysis (PCA) [32] demonstrates tight clustering of biological replicates and related samples and reveals ample differences between populations and conditions (Fig. 2). The first principle component accounts for 23 % of the total variance and clearly separates the different conditions with the exception of RT and CS. The second principal component separates the African from the European samples and accounts for 18 % of the total variance. Since the Dutch and the Zimbabwean strain strongly resemble the Swedish and Zambian strains, respectively, we treat all European and all African strains each as a single population in most subsequent analyses. We, however, performed all of these analyses also with only the Swedish and Zambian lines (results not shown) without strong effects on the outcome.

### Verification of RNAseq by qPCR

For eight genes (*Frost*, *Hsp23*, *brinker*, *smp30*, *TotA*, *TotC*, *CG10912*, *CG12164*) we performed qRT-PCR experiments in one European (SU08) and one African (ZI418) strain on a finer timescale: before the cold shock, 3.5 h into the cold shock, right at the end of the 7 h cold shock, 15, 30, 45, 60, 90, 120, and 240 min after the cold shock. Overall, the RNAseq- and qRT-PCR-results are in very good agreement (Additional file 2: Table S2). Thus, we are confident that our measurements of gene expression are accurate. Additionally, we also took samples after the timepoint of 50 % recovery (20 + 6 and 43 + 12 min recovery + handling time for SU08 and ZI418, respectively), separately for recovered and unrecovered flies. Notably, expression of all eight genes was virtually independent of recovery status. Data for the three most informative genes *Frost*, *Hsp23*, and *CG10912* is depicted in the (Additional file 3: Figure S1).

### Global expression differences between conditions

In order to identify the common properties of the cold shock response, we compared the numbers of mapped reads for each gene in the three cold treatments (CS, rec15, rec90) to the respective numbers at RT across all eight strains. For CS, in general, only minute changes in gene expression are apparent, which probably reflects the strong reduction in overall transcription at ~0 °C. Still, we identify 38 genes at a 5 % FDR-cutoff that show consistent, if only moderate downregulation (Additional file 4: Table S3). These genes are functionally enriched for being involved in the (negative) regulation of cellular metabolism and for being located in the nucleus. Prominent examples hereof are the genes hairy and *extramacrochaetae*, which are named according to their role in bristle patterning, but play a part in a wide variety of physiological and developmental processes via protein dimerizing with a range of transcription factors and thus abolishing their DNA binding capability [33]. In contrast, not a single gene is found to be upregulated at the CS timepoint.

The most striking characteristic of the cold shock response in the recovery phase is the swift and massive increase in the expression of molecular chaperones. Already 15 min after the end of the cold shock we find several heat shock proteins (Hsp) to be strongly upregulated compared to their expression at RT. This is in line with similar findings in previously published studies (e.g. [27]) and equally true for African and European flies. The gene with the highest fold-change is *Hsp70* (we treat the six copies of *Hsp70* as a single gene, see also Methods, Ambiguous mapping) with a roughly 60-fold increase in expression at rec15. This is accompanied by a more than 4-fold upregulation of *DNAJ1*/*Hsp40* and *starvin*, which are both known to closely interact with *Hsp70* at the protein level [34, 35]. In total, 364 and 518 genes are significantly up- and downregulated, respectively, at rec15 (Additional file 5: Table S4 & Additional file 6: Table S5). For these gene sets, the majority of GO enrichment found at a significance cutoff of 5 % is for the set of upregulated genes. Here, a few stress/stimulus response terms, which mostly are driven by Hsps, and a few broader terms related to regulation and development are enriched in the category “biological process”. For the downregulated genes the only significantly enriched GO term is “RNA export from nucleus”.

At rec90, 1535 genes are higher expressed than at RT and 1979 genes are less expressed (Additional file 7: Table S6 & Additional file 8: Table S7). Again, many genes that are highly upregulated belong to the Hsp gene family and the list is topped by *Hsp70* with a more than hundredfold increase in expression compared to RT. Besides molecular chaperones, notable examples of the utmost upregulated genes are *Frost*, which has been identified for being one of only few known genes that respond strongly towards a cold- but not a heat-shock [36], and a couple of genes involved in immunity (see also Discussion), e.g. *Jun-related antigen* [37], *Drosomycin-like 2* [38], and *Cecropin C* [39]. Overall, the upregulated genes are enriched for a wide variety of often broad GO terms, including regulation, localization, response to stimulus, immune response, (protein) binding, plasma membrane, cytoplasm, cell cortex and junction, cytoskeleton and ESCRT complex. The downregulated genes, on the other hand, are enriched for e.g. oxidation-reduction, lipid metabolism and intracellular membrane bounded organelle. Interestingly, four of the 16 genes with a log2 fold-change (L2FC) significantly below −1 contain a major facilitator transmembrane transport domain. Another three of these 16 genes have a poorly characterized domain of unknown function (DUF-227), which based on sequence similarity might be involved in the deactivation of ecdysteroid growth hormones.

### General expression differences between populations

In order to identify general characteristics of differentiation between the continents, we compared the numbers of mapped reads for each gene between European and African samples across all experimental conditions. We find 3486 genes with a significantly higher expression in Europe (Additional file 9: Table S8) and 3440 genes with a significantly higher expression in Africa (Additional file 10: Table S9), meaning that almost 50 % of all genes are differentially regulated due to continental origin. The heavily Europe-biased genes include well known examples as *Cyp6g1* [40] and *Cyp6g2* [41], which are involved in insecticide resistance. *Cyp6g1* is consistently about fourfold upregulated in Europe in our study and in two other transcriptomic studies [17, 18], in both of which it is the gene with the strongest overexpression in Europe. Overall, the Europe-biased genes are GO enriched most prominently for terms related to protein biosynthesis. A GO analysis of the Africa-biased genes, in turn, reveals, for instance, an overrepresentation of genes that play a part in development, regulation, binding, and/or belong to the nucleus.

### Genotype by environment interactions

Overall, the transcriptional cold shock response is fairly similar in Europe and Africa. There is not a single gene that is at the same time significantly upregulated due to the cold shock in one population and downregulated in the other. Furthermore, almost all genes that respond strongly towards the cold shock do so in a similar fashion in both populations. Looking for a statistical interaction between the effects of origin and condition, we identify 16 such genotype-environment-interaction (GEI) genes (Table 1). Two of those, namely *HR38* and *CG44247*, display GEI for rec15 vs. RT. In both cases expression first decreases at rec15 in the European population before it increases at rec90. In the African population, on the contrary, expression gradually increases after the cold shock. At RT and rec90 the two genes are similarly expressed in both populations. The other 14 GEI genes exhibit a population specific regulation for rec90 vs. RT. For six of these genes the L2FC is smaller in Africa and for eight genes larger. Interestingly, for 14 and 15 of the 16 GEI genes the absolute extent of the cold-induced change in expression is greater in the African population at rec15 and rec90, respectively (Table 1). There is only a single gene (*cwo*) for which the absolute change in mRNA abundance is substantially larger in Europe.

**Table 1:** Genes with significant genotype by environment interaction

Gene	Significance of interaction FDR(BH)	L2FC rec15 vs. RT Europe	L2FC rec15 vs. RT Africa	L2FC rec90 vs. RT Europe	L2FC rec90 vs. RT Africa
*HR38*	4.04E-02^a^	**−0.21**	**0.59**	**3.10**	**3.24**
*CG44247*	4.05E-02^a^	−0.26	0.24	**0.41**	**0.55**
*GATAe*	9.71E-04	**−0.11**	**−0.61**	**−0.36**	**−1.63**
*CG13607*	1.06E-03	**−0.40**	**−0.72**	**−0.83**	**−2.67**
*CG11897*	1.09E-03	**−0.06**	**−0.35**	**−0.10**	**−0.69**
*CG11741*	1.99E-02	**−0.74**	**−1.03**	**−0.62**	**−1.86**
*cwo*	1.41E-02	**−0.17**	**−0.44**	0.81	0.04
*rudimentary*	1.63E-02	**0.07**	**−0.31**	**0.08**	**−0.44**
*wunen*	7.82E-06	**0.04**	**0.18**	**0.49**	**1.14**
*brummer*	1.33E-04	−0.19	0.15	**0.39**	**1.26**
*CG18744*	4.51E-04	**0.43**	**1.29**	**0.93**	**2.29**
*CG7017*	1.73E-03	**−0.25**	**0.41**	**0.93**	**3.75**
*Lnk*	3.24E-03	**−0.04**	**0.12**	**0.14**	**0.69**
*CG15126*	1.12E-02	**0.17**	**0.48**	**0.86**	**1.66**
*CG13482*	1.99E-02	**0.70**	**1.25**	**1.22**	**2.55**
*GstE8*	4.96E-02	**0.11**	**0.50**	**0.49**	**1.42**

Significance of interaction applies to rec90 vs. RT except noted otherwise

Bold L2FC values indicate greater absolute L2FC for the African population

^a^Interaction is significant for rec15 vs. RT

### Genome-wide canalization of European gene expression

**Fig. 3:**
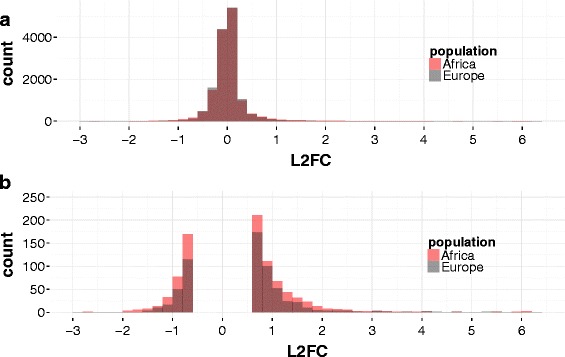
Genome-wide L2FC per population. Using DESeq2 [42] the log2 fold-change (L2FC) in expression between rec90 and RT was calculated for 13803 genes with sufficient read count in both populations separately for African and European flies. Genes were then grouped into distinct bins according to their L2FC. Bin size is 0.2. The area where the African and European histograms overlap is depicted in dark red. **a** All genes, **b** genes with an absolute L2FC > 0.6

**Fig. 4:**
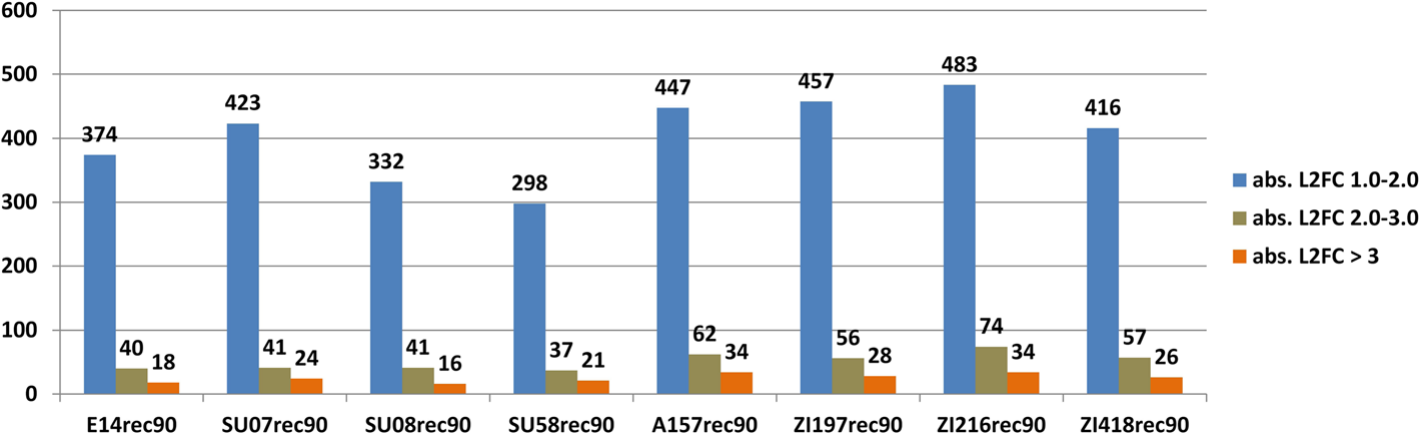
Amount of highly plastic genes per strain. Genewise L2FC between rec90 and RT was calculated for each strain after normalization of raw counts using the TPM method [43, 44]. All genes with zero read count in one sample were excluded resulting in 12617 genes in total. Depicted are only genes with an absolute L2FC > 1 with no respect to up- or down-regulation

To further explore this pattern on a genome-wide scale, we computed the cold-induced L2FC in expression (rec90 vs. RT) for all genes with sufficient read count in both populations (*n* = 13803 genes) separately for African and European flies using DESeq2 [42]. Genes were then grouped into distinct bins with a width of 0.2 according to their L2FC (Fig. 3). Bins range from extreme downregulation to no change in expression to strong upregulation. The majority of genes exhibit only minor changes in gene expression and the amount of up- and down-regulated genes is comparable, resulting in an approximately normally distributed histogram in both populations. In the overlay of the histograms for the European and the African population, however, one can see that in the European population bin size is relative to the African population larger for bins with a small absolute L2FC and smaller for bins with a high absolute L2FC (Fig. 3). In other words, more genes show a particularly strong cold-induced up- or downregulation in the African population. Thus, gene expression is canalized across control and post cold shock conditions in Europe. To corroborate these findings, we additionally verified this pattern in the individual strains. In this case, we calculated the genewise L2FC for each strain after normalization of read counts by the TPM method [43, 44] for all 12617 genes with at least one read in every sample. In all four African strains the amount of genes with a high absolute L2FC between rec90 and RT exceeds the numbers in all four European strains (Fig. 4). For the expression change between rec15 and RT this pattern is much less clear. Still, numbers of genes with an absolute L2FC > 1 are on average higher for the African strains.

### Functional validation of candidate genes via gene knockdown

It has been previously reported that an RNAi induced single gene knockdown of *Frost*, *Hsp22*, and *Hsp23* measurably diminishes cold tolerance [25, 26]. However, an effort to replicate these results for *Frost* was unsuccessful [30]. Following their experimental approach, we knocked down 48 individual genes using the VDRC RNAi library [29] and two ubiquitous driver lines (αTub and Act5C). Twenty-two of the 48 single gene knockdowns, including *Frost*, *Hsp22*, and *Hsp23*, are viable in both sexes without any apparent morphological abnormalities (Additional file 11: Table S10). However, neither for *Frost*, nor for *Hsp22*, nor for *Hsp23* does the knockdown markedly increase CCRT or decrease cold shock survival, contrary to what had been originally reported [25, 26]. For the gene *Frost* we additionally monitored CCRT and cold shock survival after cold shocks of varying lengths (7 h, 9 h, 12 h, 18 h) and for flies aged between 1 and 12 days. Despite expression of *Frost* being reduced to roughly 10 % in the knockdown cross compared to the control (as verified by qPCR), both phenotypic measures are virtually unaffected. In a similar fashion, the knock down elicits no measurable clear-cut change in CCRT and cold shock survival for the other 19 less extensively tested genes, despite some of these genes being strongly upregulated in response to the cold shock or belonging to the GEI-gene set.

## Discussion

We have measured the genome-wide transcriptional response towards a cold shock in temperate European and tropical African populations of *Drosophila melanogaster* via RNA sequencing. Our data shows, first of all, that there is very little change in expression during the cold shock, which is probably due to a general halt of transcription at 0 °C. Secondly, already 15 min after the end of the cold shock several hundred genes are differentially expressed compared to RT and after 90 min of recovery this number further increases to encompass roughly a third of all genes. Many of the genes that are most strongly upregulated belong to the heat shock protein family or to other classes of molecular chaperones. Thirdly, despite ample population differences in baseline expression, the cold shock response appears fairly similar in both populations, with only 16 genes showing a statistical interaction between the effects of origin and condition. It is precisely this kind of genotype-environment interaction (GEI) that is considered to be the hallmark of local regulatory adaptation. Interestingly, the great majority of these GEI-genes exhibit a stronger extent of cold-induced change in the African population. A similar pattern is visible on a genome-wide scale, where numbers of genes with exceedingly sharp up- and downregulation in response to the cold shock are much higher in Africa.

### Decreased thermosensitivity of gene expression in temperate flies

Ninety minutes after the cold shock (and to a lesser extent also 15 min after the cold shock) the genome-wide cold-induced change in expression is considerably smaller in the cold-tolerant European population. While the direction of transcriptional change for a given gene, i.e. up- or downregulation, is mostly the same in the two populations, the extent of the cold-induced change is often larger in the cold-sensitive African population (Fig. 3). This pattern of canalization of gene expression in temperate flies has been observed in other studies in *D. melanogaster* [45–47] and there is evidence that it is under positive selection [46]. On the other hand, extreme cold sensitivity has been associated with an exaggerated transcriptional stress response [21]. Accordingly, exposure to zero degrees, which is a novel stressor for the African flies, might elicit an overshooting stress response or disturbance of regulatory networks, while European flies have adapted to better maintain certain optimal transcript levels. We have chosen the rec90 time point, because in preceding qPCR experiments (Additional file 3: Figure S1) and in the literature [25–27] some prominent stress response genes peak in expression around this timepoint. This is true for both European and African flies and there is no evidence that this peak in expression generally occurs later in the African population and that thus the canalization pattern at this time point is solely the result of a timing shift. Though even if this would be the case, the faster return towards baseline expression patterns in the European flies might still present a case of canalization and be potentially adaptive. It is, however, difficult to directly relate these regulatory differences to differences in CCRT based on the expression data alone. Most parts of the transcriptional cold shock response are likely not overly relevant for chill coma recovery. Rather, faster chill coma recovery and canalization of gene expression of European flies are both phenotypic characteristics of cold adaptation. It would require additional timepoints in the recovery phase to identify individual genes whose expression directly follows the particular chill coma recovery dynamics in the different populations.

### Transcriptional change during the cold shock

We find several genes with GO terms related to the (negative) regulation of metabolism/transcription to be downregulated during the cold shock. Since de novo transcription in *D. melanogaster* is apparently very limited at 0 °C as the transcriptomic data suggests (see also [27, 48]), this pattern might be caused by active and specific RNA degradation, which remains possible at very low temperatures [49]. The downregulation of genes that play a part in regulatory processes in the nucleus may represent a preparation for the burst of gene expression in recovery phase. However, due to the small change in expression of all of these genes, further work is required to confirm these findings, possibly after a prolonged cold shock to allow for the accumulation of slow changes.

### Chaperones, the cytoskeleton and the stabilization of membranes

The cold shock response in the recovery phase is characterized by the massive upregulation of molecular chaperones. These proteins bind to other proteins and are responsible for the reversion of undesirable conformational changes induced by stressors and assist in the degradation of irreversibly misfolded proteins [50]. Several of these chaperones, most prominently members of the heat shock protein family, have previously been shown to be strongly upregulated after a cold shock in *Drosophila* [19, 27] and other insect species [51, 52]. The chaperonin-containing T-complex (CCT) is a ring-shaped complex, which consists of eight different subunits and is involved in the folding of nascent cytoskeletal proteins [53]. The cytoskeleton plays an important role in cold hardening in plants [54] and cytoskeletal genes have been shown to be upregulated in several insects after cold exposure [51, 55, 56]. In pupae of the onion fly *Delia antiqua* high mRNA levels of CCT genes correlate with cold hardiness and prevent actin-depolymerization, thus stabilizing the membrane [57]. We find all eight subunits of the CCT to be strongly upregulated at rec90. Additionally, we see an upregulation of the major component of the actin cytoskeleton *Actin5C*, of all eight subunits of the Arp2/3-complex, which is responsible for Actin-polymerization and branching, and of *rhea*, which is responsible for the anchoring of the cytoskeleton in the plasma membrane. Altogether, this has the potential to strengthen the cytoskeleton and its connection to the membrane and hence to increase membrane stability and to prevent extensive ion leakage. Besides, the actin cytoskeleton takes part in intracellular protein transport [58], which might be in particular demand after the cold shock to get rid of denatured proteins and to provide for repair and structural adjustments. The importance of protein degradation is further corroborated by an upregulation of the endosomal sorting complex required for transport (ESCRT) [59], which is GO enriched among the upregulated genes at rec90. Again, processes related to vesicular transport have been found to be upregulated in response to a cold shock in other insects as well [51].

### The expression of immune genes in response to the cold shock

In *D. melanogaster* the response towards a cold shock has been associated with an increased expression of immune related genes [20, 21] and there are various hypotheses why this might be the case [60]. In our study we find a significant enrichment of the GO term “response to biotic stimulus” for the 120 genes that are consistently more than twofold upregulated at rec90. The 11 genes that drive this pattern are *Hsp27*, *Relish*, *ets at 21c*, *pdgf- and vegf-receptor related*, *poor imd response upon knock-in*, *Drosomycin-like 2*, *unc-45*, *daughter of sevenless*, *unpaired 3*, *pancreatic eif-2alpha kinase*, and *Cecropin C*. Likewise, several immune related terms are GO enriched among all upregulated genes at rec90. The upregulation of immune related genes is on average marginally stronger in the African population, but it is often very inconsistent among strains and even among biological replicates. The flies that were used for the generation of the transcriptomic data were healthy in appearance, but they were grown under standard lab conditions and not in a sterile environment, so we cannot rule out differences in microbial load and other factors that might influence the immune system. Still, our data suggests considerable crosstalk between the cold shock response and the immune system, albeit for several genes in a somewhat erratic fashion.

### Conserved patterns of the transcriptional cold shock response

Gene expression studies that try to answer very similar questions using different biological material and/or different methodologies often arrive at vastly different results. Patterns that are conserved despite of these minor experimental differences, are likely more reliable and relevant in nature. Here we compare our results with a few studies that examine the transcriptional cold shock response using different fly populations, experimental approaches and means of quantifying gene expression [19, 20, 27, 48]. Qin et al. [19] measured the change in gene expression 30 min after a 2 h cold shock via microarrays in 5-7d old males. They identify 31 upregulated genes, grouped into five functional categories: stress response, membrane, mitochondrial and energy, expression, and other. Whereas all five stress response genes (*Hsp83*, *Hsp 26*, *Hsp23*, *Ubiquitin-63E*, and *Frost*) are also upregulated in our study at rec90, only 11 of the remaining 26 genes including *CG10912* show this pattern as well. Of the six downregulated genes [19], three show a downregulation at rec90 including *smp-30*. Sinclair et al. [48] monitored expression of five genes, namely *Frost*, *smp-30*, *Hsp23*, *Hsp70* and *Desat2* during a 3 h cold shock and in the subsequent 3 h recovery period in 5d old males. They see no change in expression during the cold shock, but an upregulation for *Frost* and *Hsp70* and a downregulation for *smp-30* in the recovery phase. In contrast to our findings, they observe no upregulation of *Hsp23* and a slight increase in the expression of *Desat2*, which is strongly downregulated after the cold shock in our study. In agreement with Colinet et al. [27], who subjected 4d old virgin male flies to a 9 h cold shock, we find all Hsp to be strongly upregulated in response to the cold shock, with the exception of *Hsp60* and *Hsp67*, which consistently show only relatively weak upregulation. Zhang et al. [20] subjected virgin females to three different cold treatments with short (2 h CS), prolonged (10 h CS) and repeated (2 h CS on 5 consecutive days) exposures to cold, which results in largely non-overlapping gene expression changes 6 h after recovery. They find only three genes (*TotA*, *hephaestus*, *CG11374*) to be upregulated in all three treatments. Of these *hephaestus* is the only gene to be upregulated in our study, albeit only to a marginal extent (L2FC rec90 vs. RT = 0.10) and with a FDR slightly above 5 % (0.079). Expression of *CG11374* remains relatively constant over the different conditions in our experiment, whereas the expression of *TotA* is characterized by huge variations between biological replicates and, thus, likely influenced by factors other than the cold shock. The same is true for the other members of this gene family (*TotC*, *TotM*) that were also upregulated in at least one of the cold treatments in the study by Zhang et al. Of the 20 genes that are differentially regulated in two cold treatments in their study, six are likewise affected at rec90. *CG15043*, *Attacin A*, *urate oxidase*, and *Attacin B* are consistently upregulated, whereas *CG9463* and *CG15533* are strongly downregulated.

### Conserved regulatory population differences

Additionally, we compare baseline differences in gene expression with three studies that likewise assess regulatory differences between African and European populations in whole male [17, 61] and female [18] flies. Performing differential expression analysis on our RT samples only, i.e. on unstressed flies for a dataset encompassing eight samples per population, we find 867 Europe-biased and 793 Africa-biased genes at a 5 % FDR cutoff. The only study that uses an identical technical framework, i.e. RNAseq plus the same mapping procedure and genome annotation, is Paparazzo et al. [61]. They have performed RNAseq in threefold biological replication for mass bred Dutch and Zimbabwean populations that were generated by outcrossing 12 Dutch (including E14) and 10 Zimbabwean (including A157) inbred lines. Their baseline controls, however, may technically not have been completely unstressed, since stripes with oil were inserted into the fly vials (see [61] for details). For these six samples we identify 206 Europe-biased and 322 Africa-biased genes. Only 27 (13.1 %) and 25 (7.8 %) genes are significantly overexpressed in Europe and Africa, respectively, in both our and their dataset. The 27 shared Europe-biased genes include *Cyp6g1*, *Cyp6g2*, *Cyp6t3*, and *Cyp313a1*, all of which belong to the Cytochrome P450 gene family and are implicated in the response to insecticides. An example of the shared Africa-biased genes is *Amyrel*. Furthermore, 17 genes are differentially expressed in an opposite fashion in the two datasets. The comparison between our results and Hutter et al. [17] and Müller et al. [18] is hampered by technical differences, since these two studies use microarrays and different genome annotations. Thus, we first generated datasets that only include genes that are represented in each study. For the comparison of our results with Hutter et al. [17] this encompasses 4,500 genes, for the comparison with Müller et al. [18] 5,216 genes, and for a comparison between all three datasets 2,354 genes. Both studies compare gene expression differences between several (8–12) Dutch and Zimbabwean inbred lines. For Hutter et al. [17], eight of the 74 genes (10.8 %) overexpressed in Europe overlap with our dataset, including *Cyp6g1*, *CG9509*, and *Malic enzyme*. Twenty-three of 85 genes (27.1 %) are, on the other hand, consistently upregulated in Africa. Interestingly, the sole gene with an opposing expression pattern is *CG10912*, which is strongly upregulated in response to the cold shock in our data and other studies [19, 20]. This gene is Africa-biased in [17] and Europe-biased in our data. For Müller et al. [18], 19 of 312 genes (6.1 %) are Europe-biased in our dataset and 15 of 244 genes (6.1 %) are Africa-biased in our dataset, whereas 31 genes show an opposing pattern. The smaller proportion of overlapping genes and the higher amount of genes that display differential expression in opposite directions when comparing our data to Müller et al. [18] relative to comparing it with Hutter et al. [17] is likely owed to sex-specific differences in gene expression. *Cyp6g1* is the only gene that is overexpressed in Europe in both of these studies and our dataset, whereas two genes, namely *Actin 88 F* and *retinin*, are consistently Africa-biased. If we include the study by Paparazzo et al. [61] *Cyp6g1* and *retinin* are the sole genes to be overexpressed in Europe and Africa, respectively, in all four studies. While expression of *Cyp6g1*, which is also overexpressed in European *D. simulans* [62], is associated with DTT resistance [40], *retinin* is a cornea-specific protein and likely secreted into extracellular space [63]. Overall, the amount of genes that show a consistent pattern with regard to regulation differences between Europe and Africa is considerably small.

### Phenotypic effects of an RNAi induced single gene knockdown

It has in general been very challenging and often unsuccessful to link differences in gene expression with phenotypic changes. In this study we evaluated the functional consequences of a single gene knockdown on cold tolerance for 22 individual genes, for which the knockdown was viable (Additional file 11: Table S10). Our approach was aimed at detecting relatively large and general effects. Although the knockdown reduces expression roughly tenfold, it elicits no clear-cut phenotypic effect on cold tolerance for any of the 22 genes, though of course this is by no means a proof that there is no such effect. CCRT and other cold tolerance related traits are highly plastic, intrinsically variable and presumably multigenic. Therefore, the effect size of a single gene knockdown will often be very small and may vary due to minor environmental fluctuations and/or in a random fashion. Additionally, many of the investigated genes belong to multigene families and, thus, might be at least partially redundant in function. Furthermore, CCRT and cold survival are just proxies for cold tolerance. Changes in gene expression that do not influence these particular traits might very well still be relevant for cold tolerance. The fact that we were not able to replicate the previously reported phenotypic consequences of a knockdown of *Frost*, *Hsp22*, and *Hsp23* [25, 26], despite closely following the outlined experimental procedure, might be attributed to minor differences in fly culture or a different genomic background due to different driver lines used for the knockdown crosses. In any case, our findings together with those of Udaka et al. [30], who likewise observe no such effect on cold tolerance after knocking down expression of *Frost*, suggest that the reported phenotypic implications of altered transcript levels of these genes are not general. Moreover, the lack of any clear-cut effect of the gene knockdown for all 22 analyzed genes further strengthens the notion of cold tolerance as a multigenic phenotype with presumably minor effect sizes for individual genes.

### Limitations of this study and perspectives

Though we have analyzed the genome-wide transcriptional response towards a cold shock at three different timepoints and in natural fly populations of vastly differing cold tolerance in extensive replication, our study remains limited with respect to several aspects. First, we have employed only male flies to avoid the impact of pregnancy while at the same time not being forced to separate virgin females early on. There are, however, huge regulatory differences between the sexes [18, 64]. Thus, results from male flies cannot easily be generalized. Additionally, we do not cover the topic of reproductive diapause [65], which is considered to be crucial for overwintering and population persistence in temperate environments. Secondly, we have extracted RNA from whole flies since there is no conclusive indication for a particular tissue to be of primary importance in the cold shock response and since the cold shock should in principle affect the whole fly. Nevertheless, the cold shock response might vary greatly between tissues, even in opposite directions, and this might partly obscure our results. Thirdly, we have measured the transcriptional response towards a single 7 h cold shock without any acclimation pretreatments. Different cold shock durations and repeated cold shocks with intermittent recovery periods, however, elicit varied gene expression changes [20]. Moreover, cold rearing and preceding exposures to cold greatly alter cold tolerance related phenotypes (Additional file 1: Table S1) [12, 66] and patterns of gene expression [45, 67]. Hence, in order to obtain a more comprehensive picture of the cold shock response and of regulatory population differences that form the basis of cold adaptation, it is necessary to evaluate the cold-induced changes in gene expression in both sexes, different life stages, and for individual tissues over an extensive set of preferably natural conditions. Finally, a common challenge that stress-related transcriptomic studies on sensitive and tolerant organisms face is the difficulty to discern if deviating patterns of stress-induced gene expression in the tolerant organism are in itself adaptive or simply the result of reduced intrinsic stress. This distinction is particularly complicated since functional evidence is often hard to come by.

## Conclusions

Rather than identifying individual genes with vast regulatory population differences, we detect relatively subtle population differences over a wide range of genes. The salient pattern, in this respect, is that many more genes respond strongly towards the cold shock in the cold sensitive African population. The ability to maintain favorable expression levels can be key to cope with extreme environmental fluctuations [6], since plastic changes induced by novel environments are often non-adaptive [68]. In *D. melanogaster*, gene expression is known to be more canalized in temperate populations [45–47]. Furthermore, an exaggerated stress response has been associated with extreme cold sensitivity [21]. Overall, our results further highlight the importance of canalization of gene expression for cold adaptation and emphasize its polygenic nature. On the other hand, however, phenotypic plasticity is the major factor influencing cold tolerance [12] and plastic responses to cold conditions, which also involve changes in gene expression [45, 67], appear to be adaptive at large even in tropical populations (Additional file 1: Table S1) [12]. Thus, special consideration should be directed towards the expression patterns that remain relatively conserved over a wide range of environmental conditions in temperate compared to tropical populations.

## Methods

### Fly strains and culture

The eight fly strains whose transcriptomes were analyzed in this study, consist of three Swedish (Umea, collected in 2012 by R. Wilches and S. Laurent, unpublished results), three Zambian (Siavonga, collected in 2010 by R. Corbett-Detig, [23]), one Dutch (Leiden, collected in 1999, [69]), and one Zimbabwean (Lake Kariba, collected in 1994 by T. Mutangadura, [70]) strain. The same populations, but many more individual strains (~30) for Sweden and Zambia were also used for the initial phenotyping experiments. All strains are isofemale and their heterozygosity has been further reduced due to at least 10 rounds of full sib inbreeding. Flies were cultured under standard lab conditions at 22 °C ± 1 °C, 25–50 % air humidity and a 14 h/10 h light/dark cycle on a sugar beet molasses and cornmeal medium containing agar-agar for medium consistency, dry yeast for oviposition promotion and propionic acid and nipagin as preservatives.

### Chill coma recovery assays

To quantify cold tolerance we used CCRT as a metric [11]. Briefly, CCRT is defined as the time it takes for a fly to recover, i.e. to stand upright on its six legs, after being brought back to room temperature following a cold-induced coma. Sorting and sexing of flies prior to the experiments was conducted using CO_2_-anesthetization. Flies were always allowed to recover in food vials for at least 24 h before the onset of the cold shock. In the initial phenotyping assays age of flies was 2–5 days, for subsequent analyses flies were always 5 days old. For the cold shock, they were transferred to empty plastic vials, immediately put into an ice-water bath and kept in the dark for the entire duration of the cold shock. Mostly, experiments were conducted with ~10 flies per experimental vial. Recovery status was monitored in 5 min intervals at ambient temperatures (22 ± 1 °C). CCRT was averaged over multiple vials from different experiments. Since female and male recovery time was almost identical, data from the two sexes was pooled. Population-wide CCRT values were averaged over the CCRT of all individual strains tested. For the analysis of phenotypic plasticity we reared flies additionally at 17 °C and/or included an acclimation treatment of 12 h at 6 °C before the onset of the cold shock.

### Sample preparation, RNA extraction and RNA sequencing

Extractions were performed in January and February of 2014. Samples were obtained at four distinct conditions: Without cold treatment at room temperature, 3.5 h after beginning of the cold shock, and 15 and 90 min after flies have been brought back to room temperature following a 7 h cold shock at 0 °C in an ice-water bath. For every strain-condition-combination two biological replicates were produced roughly 2 weeks apart and with flies stemming from different vials to account for vial effects. For every sample 16 male flies at 5 days of age were frozen in liquid nitrogen. Nucleic acid extraction was performed using Epicentre MasterPure Complete DNA and RNA Purification Kit according to the manufacturer’s protocol without DNAse treatment. Sample quality was assessed using Nanodrop (Thermo Fisher Scientific) and Bioanalyzer (Agilent Technologies). All samples were free from considerable amounts of contaminants and showed no signs of RNA degradation. Samples were stored at minus 80 °C prior to shipment to a sequencing company (GATC Biotech, Konstanz, Germany) for PolyA-enrichment, random primed cDNA synthesis, library preparation and sequencing on an Illumina HiSeq2000 yielding > > 20 million 51 bp single reads per sample with an average Phred score above 30 for the bps with lowest quality.

### Read mapping

The obtained reads were mapped to the *D. melanogaster* transcriptome (FlyBase release 5.57 [31]) using NextGenMap [71]. For every read, only the first best hit was counted. All reads mapping to multiple transcripts of a single gene were collapsed. The per sample library size ranges from 16 to 37 million reads. Average library size is 25.4 million reads. All reads that did not map to any transcript were then mapped to other features of the *D. melanogaster* genome. The reads that did not map to any of these as well were considered unmapped.

### Ambiguous mapping

With special regard to *Hsp70*, which is the gene with the highest cold-induced fold-change in expression, it must be noted that there are actually six different copies of *Hsp70* annotated in FlyBase 5.57. It would, however, require sophisticated approaches to be able to tell them apart due to their extremely high sequence similarity. Thus, they are commonly treated as a single gene in studies of gene expression [27, 48]. In our overview tables (S3–S7), though, the six copies are individually listed. The issue of ambiguous mapping further extends to other gene families and virtually to any two genes that share stretches of identical transcript sequence. Since we are not primarily concerned with within-gene-family-differences in expression and since this should affect both populations in a similar fashion, we assume this to be of little consequence for our overall results.

### Calling of differentially expressed genes and enriched gene properties

Differentially expressed genes (DEG) were called using the DESeq2 package (version 1.6.3) [42] for R (version 3.2.1) [72] and a 5 % FDR cutoff based on Benjamini-Hochberg adjusted P-values [73]. We used a model with three factors, namely continent (Europe, Africa), condition (RT, CS, rec15, rec90), and an interaction term between the two. Thus, there are eight samples per continent-condition-combination, consisting of 4 strains in twofold biological replication. For the multilevel contrasts of the factor condition (RT vs. CS, RT vs. rec15, RT vs. rec90) we performed the Benjamini-Hochberg-correction over all P-values of the three tests combined. The number of significant genes was maximized by applying independent filtering [74] with the mean expression over all 64 samples as independent filter criterion. The same correction approach was also applied to the multilevel interaction contrasts.

GO term enrichment was calculated using GOrilla [75] against the background of all annotated genes in the genome and with regard to multiple testing (<5 % FDR) [73].

### qRT-PCR

Each sample was prepared with approximately eight male flies at 5 days of age. Four biological replicates were produced for every strain-timepoint-combination. RNA extraction was performed as described above, with the exception of an additional 1 h DNAse I treatment at 37 °C to get rid of genomic DNA. Absence of genomic DNA was determined using a highly sensitive Phusion polymerase (NEB) and a set of primers that only amplifies genomic DNA (X-01435+: 5’-TGC GAA ACA GGT ACA AGT-3’; X-01435-: 5’-GGA TTC GTG AAC GGG AAA-3’). Furthermore, for a few samples noRT controls were run in the qPCR. cDNA was synthesized using random hexamers and SuperScript II reverse transcriptase (Invitrogen) according to the manufacturer’s protocol. Gene specific primers for qPCR were created with quantprime [76]. All primers were obtained from Metabion (Planegg, Germany). The ribosomal genes *RpL32* and *RpS20* were used as reference genes [46]. Primer sequences, annealing temperatures, amplicon properties etc. are listed in the Supplements (Additional file 12). SYBR green Master Mix (Bio-Rad) was used as reaction and detection reagent on a Real-Time thermal cycler CFX96 (Bio-Rad). Every sample was assessed in threefold technical replication. Gene expression was quantified using the ΔΔCt method [77].

### Functional assessment of the knockdown of individual genes

Fly lines containing gene-specific inverted repeats (IR) were ordered from the VDRC [29] and belong either to the GD or KK library. In these lines the IR is under the control of a GAL4-inducible promoter (=upstream activating sequence (UAS)), i.e. only expressed in presence of GAL4. To activate expression of the IR and, thus, the RNAi-mediated knockdown of the respective gene, young males of the IR lines and of their respective controls (w^1118^ for the GD library and KK60100 for the KK library) were crossed to young virgin females of two driver lines that ubiquitously express GAL4 under the control of either the Act5C- (Act5C-GAL4 / CyO) or the αTub-promoter (UAS-Dicer2; αTub-GAL4 / TM3, Kr-GFP). Progeny was screened for straight vs. curly wings (Act5C) or normal vs. stubble bristles (αTub) to obtain the desired cross. The efficiency of the knockdown was determined via qPCR. The knockdown- and control-crosses were phenotyped in parallel for changes in cold tolerance. CCRT was determined as described above. For the survival assays, flies were transferred back to food vials after the recovery experiments and kept at 22 °C ± 1 °C in separate vials for each sex. The proportion of living flies was counted roughly 24 h after the end of the cold shock. Mortality clearly peaks within this period of time, as subsequent checks at 48 h revealed identical numbers of fatalities in the great majority of cases.

## Abbreviations

CCRT, chill coma recovery time; CCT, chaperonin-containing T-complex; CS, cold shock; for the samples: 3.5 h into the cold shock; DEG, differentially expressed genes; GEI, genotype by environment interaction; Hsp, heat shock protein; IR, inverted repeat; L2FC, log2 fold-change; PCA, principal component analysis; rec, recovery phase; RT, room temperature; for the samples: unstressed control; SU, population from Umea, Sweden; TPM, transcripts per million; UAS, upstream activating sequence; VDRC, Vienna Drosophila RNAi Center; ZI, population from Siavonga, Zambia

## Additional files

Additional file 1: Table S1. CCRT under varying environmental conditions (XLS 26 kb) Additional file 2: Table S2. Verification of RNAseq by qPCR (XLS 29 kb) Additional file 3: Figure S1. qPCR results for *Frost*, *Hsp23*, and *CG10912.* Samples were taken at room temperature (RT), 3.5 h into the cold shock, at the end of the 7 h cold shock and 15, 30, 45, 60, 90, 120, and 240 min following a 7 h cold shock for the fast recovering Swedish strain SU08 (*pink*) and the slow-recovering Zambian strain ZI418 (turquoise). Additional samples were taken at the time point of 50 % recovery, separately for recovered and unrecovered flies. These time points correspond to 20 + 6 and 43 + 12 min of recovery + handling time for SU08 and ZI418, respectively. Error bars denote the standard deviation over four biological replicates. (PDF 416 kb) Additional file 4: Table S3. Downregulated genes CS vs. RT (FDR < 5 %) (XLS 24 kb) Additional file 5: Table S4. Upregulated genes rec15 vs. RT (FDR < 5 %) (XLS 97 kb) Additional file 6: Table S5. Downregulated genes rec15 vs. RT (FDR < 5 %) (XLS 129 kb) Additional file 7: Table S6. Upregulated genes rec90 vs. RT (FDR < 5 %) (XLS 354 kb) Additional file 8: Table S7. Downregulated genes rec90 vs. RT (FDR < 5 %) (XLS 445 kb) Additional file 9: Table S8. Europe-biased genes (FDR < 5 %) (XLS 767 kb) Additional file 10: Table S9. Africa-biased genes (FDR < 5 %) (XLS 765 kb) Additional file 11: Table S10. Viability after ubiquitous knockdown via RNAi for 48 genes (XLS 31 kb) Additional file 12: qPCR primer sequences etc. (DOC 66 kb)
