# Supplementary figures and images for: Canalization of gene expression is a major signature of regulatory cold adaptation in temperate Drosophila melanogaster
Source: BMC Genomics. 2016 Aug 8;17:574. doi: 10.1186/s12864-016-2866-0 (PMC4977637; doi:10.1186/s12864-016-2866-0)

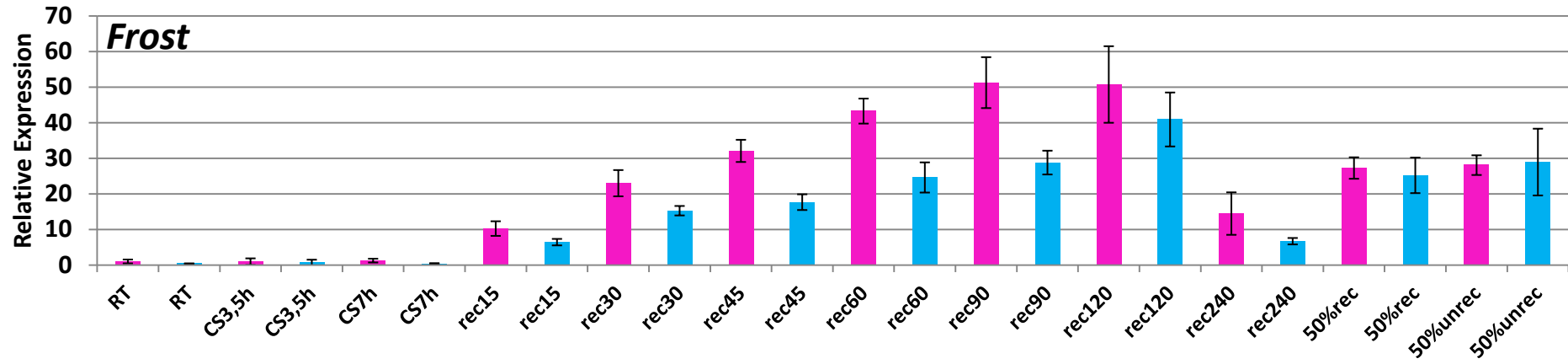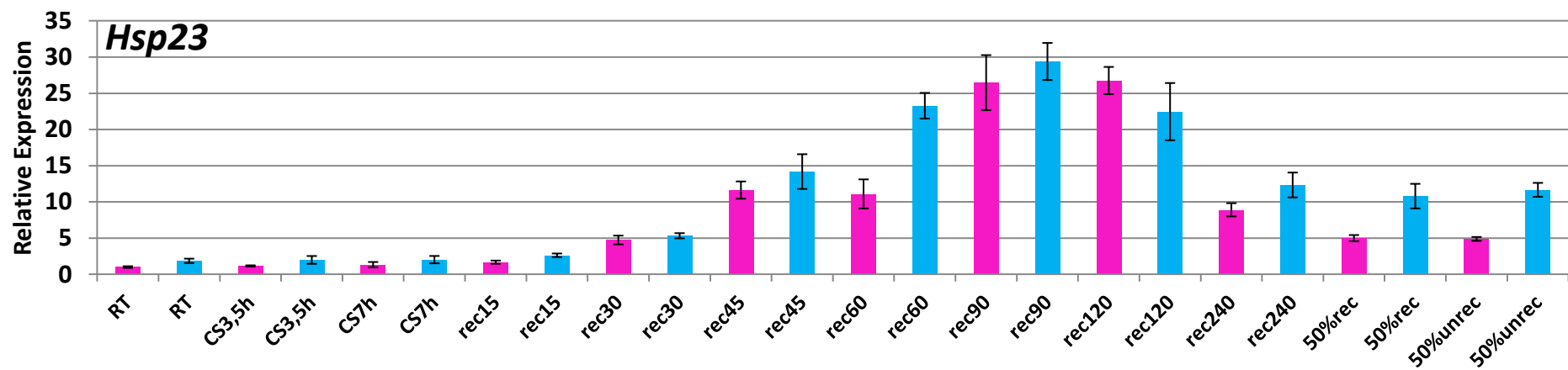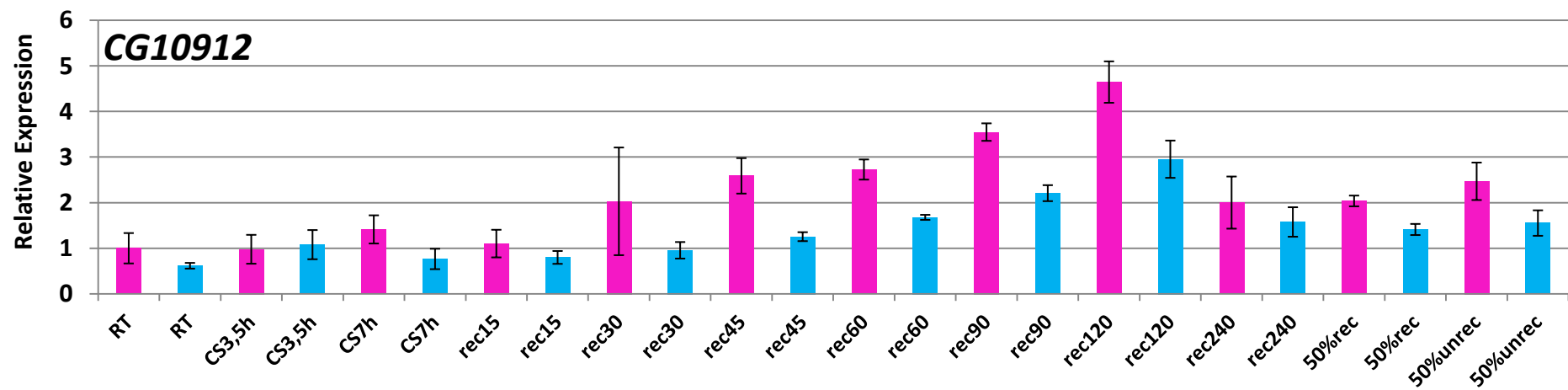

Supplement: Additional file 3: Figure S1. — qPCR results for Frost, Hsp23, and CG10912. Samples were taken at room temperature (RT), 3.5 h into the cold shock, at the end of the 7 h cold shock and 15, 30, 45, 60, 90, 120, and 240 min following a 7 h cold shock for the fast recovering Swedish strain SU08 (pink) and the slow-recovering Zambian strain ZI418 (turquoise). Additional samples were taken at the time point of 50 % recovery, separately for recovered and unrecovered flies. These time points correspond to 20 + 6 and 43 + 12 min of recovery + handling time for SU08 and ZI418, respectively. Error bars denote the standard deviation over four biological replicates. (PDF 416 kb) [file 12864_2016_2866_MOESM3_ESM.pdf]
