# Supplementary material for: Canalization of gene expression is a major signature of regulatory cold adaptation in temperate Drosophila melanogaster
Source: BMC Genomics. 2016 Aug 8;17:574. doi: 10.1186/s12864-016-2866-0 (PMC4977637; doi:10.1186/s12864-016-2866-0)
Supplement: Additional file 12: — qPCR primer sequences etc. (DOC 66 kb) [file 12864_2016_2866_MOESM12_ESM.doc]

***RpL32***

**Transcript identifier:** [**FBtr0085594**](http://www.quantprime.de/minimal.php?page=transcripts&subaction=geneinfo&identifier=FBtr0085594&organismid=355) - type=mRNA; loc=3R:complement(25870868..25871346,25871409..25871519); name=RpL32-RA; dbxref=FlyBase:,FlyBase_Annotation_IDs:CG7939-RA,REFSEQ:NM_079843; score=3; score_text=Weakly Supported; MD5=6a0343db17883d9d1e1f7f7fa4bfb5cb; length=590; parent=FBgn0002626; release=r5.17; species=Dmel;

**Forward primer**
Sequence: ATCGTGAAGAAGCGCACCAAGC (22 b)
Melting temperature: 64.6 °C
G/C content: 54.5 %

**Reverse primer**
Sequence: TTGCGCCATTTGTGCGACAG (reverse complement: CTGTCGCACAAATGGCGCAA) (20 b)
Melting temperature: 63.1 °C
G/C content: 55 %

**Amplicon**
Size: 77 b
Melting temperature: 83 °C
G/C content: 53.2 %
Optimal annealing temperature: 62.1 °C

**Alignment with transcript sequence**
*TGTCCTTCCAGCTTCAAG*ATGACCATCCGCCCAGCATACAGGCCCAAG**ATCGTGAAGAAGCGCACCAAGC**ACTTCATCCGCCACCAGTCGGATCGATATGCTAAG**CTGTC^GCACAAATGGCGCAA**GCCCAAGGGTATCGACAACAGAGTGCGTCGCCGCTTCAAGGGACAGTATCTGATGCCCAACATCGGTTACGGATCGAACAAGCGCACCCGCCACATGCTGCCCACCGGATTCAAGAAGTTCCTGGTGCACAACGTGCGCGAGCTGGAGGTCCTGCTCATGCAGAACCGCGTTTACTGCGGCGAGATCGCCCACGGCGTCTCCTCCAAGAAGCGCAAGGAGATTGTCGAGCGCGCCAAGCAGCTGTCGGTCCGCCTCACCAACCCCAACGGTCGCCTGCGTTCTCAAGAGAACGAGTAA*GCTTAAGATTCTTGAGAGTTCTTGTAACGTGGTCGGAATACACATTTGTAAACGTTAATATACCGGACTTTTAGTTAAAAAATGATGTGCGAGTGCCGAGTTCAATTGTCATTTCTGAGATTGGGATAGCAGCACCATTGATAACATGTGCATTATCTGGATGGATA*

**Specificity test results**
Overall score: Perfect
cDNA specificity: Good
Single primer specificity: Good
Amplifies genomic DNA: No

***RpS20_***

**Transcript identifier:** [**FBtr0083964**](http://www.quantprime.de/minimal.php?page=transcripts&subaction=geneinfo&identifier=FBtr0083964&organismid=355) - type=mRNA; loc=3R:join(16647901..16647995,16648140..16648295,16648524..16648613,16648854..16649078); name=RpS20-RA; dbxref=FlyBase:,FlyBase_Annotation_IDs:CG15693-RA,REFSEQ:NM_079697; score=15; score_text=Strongly Supported; MD5=63a1710bf16281ca7bfe4b62ca1c44bc; length=566; parent=FBgn0019936; release=r5.17; species=Dmel;

**Forward primer**
Sequence: TTCGCATCACCACCCGTAAGAC (22 b)
Melting temperature: 63.2 °C
G/C content: 54.5 %

**Reverse primer**
Sequence: TTGTGGATTCTCATCTGGAAGCG (reverse complement: CGCTTCCAGATGAGAATCCACAA) (23 b)
Melting temperature: 61.3 °C
G/C content: 47.8 %

**Amplicon**
Size: 76 b
Melting temperature: 82.7 °C
G/C content: 52.6 %
Optimal annealing temperature: 61.3 °C

**Alignment with transcript sequence**
*ACTTCTCTTCGTTTTCAACCAGCAAATTGACTGGTTCAGCGCTGCTCAAAATACAAAGATTTGAATATCTCGACCAGGGAAATTGCTAAATA*ATGGCTGCTGCACCCAAGGATATTGAGAAGCCCCATGTCGGCGATTCTGCCTCTGTGCACCGCATCCGCATCACCCTGACATCCAGGAACGTGCGTTCGCTGGAGAATGTGTGCCGCGACCTGATCAACGGTGCAAAGAACCAGAACTTGCGCGTCAAGGGCCCCGTGCGCATGCCGACCAAGACCC**TTCGCATCACCACCCGTAAGAC**TCCTTGTGGTGAGGGTTCCAAGACCTGGGAT**CGC^TTCCAGATGAGAATCCACAA**GCGCATCATCGACTTGCACTCGCCCTCTGAGATCGTCAAGAAGATTACCTCCATCAACATCGAGCCCGGCGTAGAGGTTGAGGTCACCATCGCCAACTAA*GATCGGCAGATGCCACATTTTTACACCTCGAAAAGTTTGGCGTGCTAATAAAAACAAAGAACTTTTTCCATACACATTTGGCCAACTTTATTGTGCGAAAATTGGAGTACT*

**Specificity test results**
Overall score: Perfect
cDNA specificity: Good
Single primer specificity: Good
Amplifies genomic DNA: No

***Frost***

**Transcript identifier:** [**FBtr0082101**](http://www.quantprime.de/minimal.php?page=transcripts&subaction=geneinfo&identifier=FBtr0082101&organismid=355) - type=mRNA; loc=3R:complement(5470851..5471874); name=Fst-RA; dbxref=FlyBase:,FlyBase_Annotation_IDs:CG9434-RA,REFSEQ:NM_079570; score=11; score_text=Strongly Supported; MD5=b48dad2474883e39785375e2730daeee; length=1024; parent=FBgn0037724; release=r5.17; species=Dmel;

**Forward primer**
Sequence: TGCAGGAACAGAGGTGGAATAGC (23 b)
Melting temperature: 62.9 °C
G/C content: 52.2 %

**Reverse primer**
Sequence: TGACCCTGACCGTTGCCATTTG (reverse complement: CAAATGGCAACGGTCAGGGTCA) (22 b)
Melting temperature: 64 °C
G/C content: 54.5 %

**Amplicon**
Size: 61 b
Melting temperature: 79.3 °C
G/C content: 49.2 %
Optimal annealing temperature: 59.5 °C

**Alignment with transcript sequence**
*GTAGTATAATCGAAACAATAGCGAA*ATGAAGGTCCTAACTCTTATCCTCTTCACTGCCTTGGTGGCAGTGGCTTCTGGCCGCCCCAATGGCGGTTGGTTCGGAAATTTTGGCAGCAACTGGGCCAGGAGGTTCGGTTGGGGCTCCGGTGACGATGAAATCG**TGCAGGAACAGAGGTGGAATAGC**CAAAATCAGTGGAATC**CAAATGGCAACGGTCAGGGTCA**GTGGGATGGCGAAAATGGTCAGGGACAATCTCACGGAAACAATCAAGGCCTCGGCCAAGGAAACGGACACGGAAACAACCATCATGGTCATCATGGCAACAGTCATGGTCATGGTAATGGTCAAGGCCACGGTGGACAGCGCCCTCCTCCACCACCACCAACGGATCTGCCTGAGTTGACCACCGAGGATGATGTGGTATCCACCACGGATGTTACCAGTCCCGCGGAGGAGACCACTCTGGCACCCGAGGTTCCAGAAGAATCTACCAGTCAGGCACCAGAGGAAATCACCACCGGCTCTGAAGAAGGCAGCGGTTCCTCGGAGGACACCACCACCCTGGCCCCTGAAGTTCCGGAGGAATCCACCACTCAGGCACCGGAGGAAAGCACCACCGACTCTGAGGATGGTAGCGGTTCTGAGGATACCACTCAGGCTCCGGAAGAAACCACCACCGAGGAGCCTGAAGAATCCACCAGCGAGGCTCCCGAAGAATCCTCCAGCGAGGCGCCCGAAGAATCCACCACAGAGGAGCCCGAAGAATCGACAACCGAGGCGCCTGTAGAATCCACAAGTGAGGCACCCGAAGAATCGACGACCGAGGCTCCCGAAGAATCCACAAGTGAGGCGCCGGAAGAATCCACTATCGATTCTTCAGCGGTCTAG*GAAGTTAGTTTCATTGTTACCAAGGTTTCTGGTTCATTTTCTCAATAATCGTTTAAAATTTGGCGTTTCCGAGTAACAAGGTTATTAAACAAAGCGTTATAAAACGAAATATGGTGCAAATATTAAACTGTTATAGCT*

**Specificity test results**
Overall score: Good
cDNA specificity: Good
Single primer specificity: Good
Amplifies genomic DNA: Possibly; putative amplicons within the following gene(s): [FBgn0037724](http://www.quantprime.de/minimal.php?page=primerpairproblem&primerpairid=16438266&string=FBgn0037724%5B%5Bprimerpair%2C 1%2C 1%2C 1%2C 23%2C 162%2C 184%2C -1%2C -1%2C 22%2C 1%2C 201%2C 222%2C u|||||||||||||||||||||||%2C u||||||||||||||||||||||%2C 61%5D%5D&type=1&organismid=355), [FBgn0012344](http://www.quantprime.de/minimal.php?page=primerpairproblem&primerpairid=16438266&string=FBgn0012344%5B%5Bprimerpair%2C -1%2C -1%2C 23%2C 1%2C 7788%2C 7810%2C 1%2C 1%2C 1%2C 22%2C 7750%2C 7771%2C u|||||||||||||||||||||||%2C u||||||||||||||||||||||%2C 61%5D%5D&type=1&organismid=355), [Unknown](http://www.quantprime.de/minimal.php?page=primerpairproblem&primerpairid=16438266&string=Unknown%5B%5Bnottested%5D%5D&type=2&organismid=355)

***Hsp23***

**Transcript identifier:** [**FBtr0076453**](http://www.quantprime.de/minimal.php?page=transcripts&subaction=geneinfo&identifier=FBtr0076453&organismid=355) - type=mRNA; loc=3L:9374984..9375867; name=Hsp23-RA; dbxref=FlyBase:,FlyBase_Annotation_IDs:CG4463-RA,REFSEQ:NM_079275; score=15; score_text=Strongly Supported; MD5=4529b00d90b0d08308ab355b2a60bde2; length=884; parent=FBgn0001224; release=r5.17; species=Dmel;

**Forward primer**
Sequence: AGCGAACTGGTGGTCAAAGTGC (22 b)
Melting temperature: 64.2 °C
G/C content: 54.5 %

**Reverse primer**
Sequence: ATCTTCGCGCTCCTCATGGTTG (reverse complement: CAACCATGAGGAGCGCGAAGAT) (22 b)
Melting temperature: 63.6 °C
G/C content: 54.5 %

**Amplicon**
Size: 69 b
Melting temperature: 84 °C
G/C content: 58 %
Optimal annealing temperature: 63 °C

**Alignment with transcript sequence**
GCGTCAGTTGAATTCAAAAAGCCAAAGCGATAACAGCTAAAGCGAAAGTAACCTATCAACAAAAGAAGTTTATTCTTTGAAGGAGAATCATCTTGAAGCAATTAAAAAACAAAAATGGCAAATATTCCATTGTTGTTGAGCCTTGCCGACGATTTGGGCCGAATGTCGATGGTGCCCTTCTATGAGCCCTACTACTGCCAGCGCCAGAGGAATCCCTACTTGGCCCTGGTTGGACCGATGGAGCAGCAGCTGCGCCAGCTGGAGAAACAGGTGGGCGCCTCGTCGGGATCGTCGGGAGCCGTGTCGAAAATCGGAAAGGATGGCTTCCAGGTCTGCATGGATGTGTCGCACTTCAAGCCC**AGCGAACTGGTGGTCAAAGTGC**AGGACAACTCCGTCCTGGTGGAGGG**CAACCATGAGGAGCGCGAAGAT**GACCATGGCTTCATCACTCGTCACTTTGTCCGCCGCTATGCTCTGCCACCCGGTTATGAGGCTGATAAGGTGGCCTCCACCTTGTCCTCCGATGGTGTCCTGACCATCAAGGTGCCCAAGCCACCGGCAATCGAGGATAAGGGCAACGAGCGCATCGTTCAGATCCAGCAGGTGGGACCCGCCCATCTCAATGTGAAGGAGAATCCCAAGGAGGCGGTGGAGCAGGACAATGGCAACGATAAGTAG*AGGACTCGTTCCGAGAGATGCCCTGCATTATTTAACCATTATCAAAGTCATACATCTGTTTTATAAGCTGTAGTTATCCAAGGACACTTCACTCATACACAATAGCCATTAAGGGTGTCCTGCTTTAATCTTAGTTTGGAATATGTATTACTAAATTGGCGAAATTAATATTACCCATAAAAATAAATAACAAGTACACTTACTTATAA*

**Specificity test results**
Overall score: Good
cDNA specificity: Good
Single primer specificity: Good
Amplifies genomic DNA: Possibly; putative amplicons within the following gene(s): [FBgn0001224](http://www.quantprime.de/minimal.php?page=primerpairproblem&primerpairid=16444582&string=FBgn0001224%5B%5Bprimerpair%2C 1%2C 1%2C 1%2C 22%2C 361%2C 382%2C -1%2C -1%2C 22%2C 1%2C 408%2C 429%2C u||||||||||||||||||||||%2C u||||||||||||||||||||||%2C 69%5D%5D&type=1&organismid=355), [Unknown](http://www.quantprime.de/minimal.php?page=primerpairproblem&primerpairid=16444582&string=Unknown%5B%5Bnottested%5D%5D&type=2&organismid=355)

***CG10912***

**Transcript identifier:** [**FBtr0086807**](http://www.quantprime.de/minimal.php?page=transcripts&subaction=geneinfo&identifier=FBtr0086807&organismid=355) - type=mRNA; loc=2R:complement(13943568..13944064,13944157..13944564); name=CG10912-RA; dbxref=FlyBase:,FlyBase_Annotation_IDs:CG10912-RA,REFSEQ:NM_137443; score=11; score_text=Strongly Supported; MD5=e1604619d9d4634145a59b096920226b; length=905; parent=FBgn0034296; release=r5.17; species=Dmel;

**Forward primer**
Sequence: TCCTGCTGGACTGCGATAAACAG (23 b)
Melting temperature: 63.1 °C
G/C content: 52.2 %

**Reverse primer**
Sequence: GCTTGGAATATGTCGGACCCTCAG (reverse complement: CTGAGGGTCCGACATATTCCAAGC) (24 b)
Melting temperature: 63.4 °C
G/C content: 54.2 %

**Amplicon**
Size: 81 b
Melting temperature: 81.3 °C
G/C content: 48.1 %
Optimal annealing temperature: 61 °C

**Alignment with transcript sequence**
*AAGTTTCAAACGAACCTCACAACGAGCAAC*ATGCAGTCACATCTCATCGCCATCCTGCTGGCCACCGTGGCCATTGGCTCCTGCATCGCGAATCCCAACCTCATCTCTGGTCCTTCGCGAATTCTTGAGATAATGAGCGCCACCAGTGATATCCAGCGTAATAATCCACAACTGACCGTAGAATGTTTTGACTACTATAATGACGTATTCAAAACCGAGTATGCGGAGTATGTGGATGAGTACAATCTGTGCGTCGATAAGTATGATGGCGGTTATGAACAGGTTCTGGAACAGTACAATTCCGTTGTCTGGGACCTCAGCAATTCCACCTTCGAATCCTGCATGT**TCCTGCTGGACTGCGATAAACAG**AATAACAGCGAAAATGCTCTATCCTGCTACTCCA**CTG^AGGGTCCGACATATTCCAAGC**AGTTGTCGAATGTGGCAGCCAATGCTTCGGTCTCGGTCAGTTCACTGCGTCAGCAGGTCGAAACACTGGTGTTCACCCGTGACCAATGCTGTTCTGCAACGTCCAGGAACTATGAGATCCGCTCCGGCGAGTCCTACGAAGATCTTCAGAAGTGCCTGAGCGGTGAGAATCCTGTGCCGGAAAGAAGCACCACCACTACCAGTTCCAGCTCCAGCTCCAGCACCAGCACCACCGCCGCCAACCCCACTACTACCACCACGACCCCATCTCCTTCAACCACCACCTCATCGTCTTCCACCTCCTCAACAACGAGATCTCCATCGACAGTTGCGCATTTTAGTTCCGTTGAAAACAGCAGTGGCAACAGCCAGCACCGATTTCCAAGAAGACTGGACAATATTTTCAAACATATTCTTTAA*GGGTTGAACAATATAAAAGTCAATATCAATAAACTAAAAAGGAACATTGCAAAAAGACA*

**Specificity test results**
Overall score: Perfect
cDNA specificity: Good
Single primer specificity: Good
Amplifies genomic DNA: No

***brinker***

**Transcript identifier:** [**FBtr0071048**](http://www.quantprime.de/minimal.php?page=transcripts&subaction=geneinfo&identifier=FBtr0071048&organismid=355) - type=mRNA; loc=X:7201974..7205645; name=brk-RA; dbxref=FlyBase_Annotation_IDs:CG9653-RA,FlyBase:,REFSEQ:NM_078514; score=15; score_text=Strongly Supported; MD5=fb8816db73f37e709498b29084c62c74; length=3672; parent=FBgn0024250; release=r5.17; species=Dmel;

**Forward primer**
Sequence: TGCGAGGACATCATCCGTCAAC (22 b)
Melting temperature: 63.3 °C
G/C content: 54.5 %

**Reverse primer**
Sequence: TCAGGTTTGTGGGCGCAGTATC (reverse complement: GATACTGCGCCCACAAACCTGA) (22 b)
Melting temperature: 63.5 °C
G/C content: 54.5 %

**Amplicon**
Size: 65 b
Melting temperature: 83.1 °C
G/C content: 56.9 %
Optimal annealing temperature: 62.2 °C

**Alignment with transcript sequence**
CAATTGTGTTTTGGATTCCTTGCCGTGCGGATCGCAAACGCGAGTTATGAGAAGAGCGTAGTAAAACGTACGAGAAGTAGCTAACAAGAAAAGGAAAAGAGCAGAAGAAGCAACAGCACGCAGACATATATTTTTTTTAACTGTGTTTTATTTATCCGATCTATACCAATCGAAATATACACAACTATATAGATTTGAAACGGATTTTGAAAAGTGCTTTCAAATAAACTCCAAAAAGTATCAGAGCGAGTGTGTGCCAGTGTGTGTATGTGAGACGCGTCGCAGTCGACGTCGCTGCTTTGGGGACAGAGCTGCCAGATCGCGAGTTATGAAAGCGCAAGCGTCGCACGCAAGTAGCAACAACAGCTACAACAAAAGCACCTAAAACTTACTACCTACTACTACTGCAAAACACACTTGTTTGACTATTATTTCGCTACATTAAAATCAACAAAAATACACACAAAACTGACCAGAATCTTGGGAAAATAAAAACAATCTTTAAAGAACGCCACGAAAATAAACCATAATCATGGATAGCAGCAGCGAACAGTTGAACGGATCGGGAGCTTTGAATTTCAAGCGGCCCAAGGATTCTTCGGAGAATGCCACAAACAGCCACACAAATAATGGCAATTCTTCGGGCAGCCCCAAAATGGGAAGTCGTCGGATTTTCACGCCCCACTTTAAGCTGCAAGTCCTCGAATCATACAGGAATGATAATGATTGCAAGGGCAATCAACGGGCCACGGCCAGGAAATACAACATTCACCGCCGGCAAATCCAAAAATGGTTGCAATGCGAGTCAAATTTGCGATCATCGGTGGCCAACAATCAGCAACAGCAGCAGCAGCAGCAACAGCAACAGCAGCAACAACAGCAGCAGCAGCAACTACTCCCACAGCAATCGGTATCGCCGACACCGGCGGTCAAGGTGTTCCATCAGCTGAGCCATCCGCTGGTGCACCAGTTGCATCACCATCACGCCGCCGCGGTGGGTCATCATCATCATCATGCCGCCCACCATCATGCCGCCCATCATCATCACGCTGCAGCAGCAGCGGCGGCAGCAGCAGCGGCAGCAGCAGCAGCAGCAGCCCACCATCATGCCGCCCACCATTTGCTGGCAGCCAATGGCATGGTTCCACACCCCCTGGCCGCCCATCCCCACTTGCATGTCCCGGTGGCAATGCATCCTCAAATGCAGCATCAGAAGGAGCAACAGCAGCAGCAGCAGCAGTTGCAGCAGGAGCAGCAGCAACAGGATCAGCAGCAGTCACAGCAGGAGACACCTGCAACCATTGCTACCAATGGCAGCAATCAGGGATCCTCGAATGTCCTTTCGGCGGCCAAAATAGCCGCTGTTGTTGCCGCCGCCATGGCCACCAGCAATGGCAATCCCACACCGACAGCAACAATCCCCGCCAGCAGCAGCAGCAGCAGCAACACATTGCCGAGCAGCAATAACAGTAGTTGCCACAACAGCAGCAGCAGCAGCAATAGCTGCAACAGCAACCAAGTGCCAATACAAGTGCCAATCCTGAGCGGATCCCCTGGGTCCACATCGTCAGCCAGTCACATTCCACATGTGCCCTTTGCCTACCATCACAATCTGCATGGCTATTTGGAGAATCGACTGGAGGCGGTTGCCACACCAGCCCCCATGGACCTCTCTCTCGGATCCTCTGCCCGCCGTCAAATGCAGTTGCACGAAAAGGATCCATCTGGTGTGGATCTCACTTTCCGCAAACGTAAGGTCATCACTAGTCCGATGCAGCCGGATAAGATTAGTAAGCTGGAGGAGGTGATCAAGAAGGAGCCCGAAACGGAAACGGAGAACGAGGATGTGGAGGTGGACGTGGAGACGGAGCAGCCGGAGGAGCACAAGCTGCCCTCTAAACAGGTCAAGCTATTTAAGCCCTATTTGCTGGACGATGATGAGGAGCAGGATCATCATCATCATCACCATCACCATCGGCAGGAGGATTTGGATGAGGGAGCTGCCGAGGAGGAGCAGGACGACGAGGAAGAGTCCCGCTATGCCGACGATGATGAGGTGGACTCCAAGGAGGCGGCCGACAAGAAACAGCGTCGCCTCAAGAAAAAGCCATCGGCAATCAACGAACAAAGGGAACCCATCATCTGGAGCAACCATCCGTATCCCGGTGGCTGTGTATCGCCCGGCAGTTCGATCACCAGCAGCTTTCAGTGCCCCACCAGTATGCAACAGCAGCAGACATTTCCCGTCGCCGGCGGGAGTCCTAATCAGCAATTTCAGGACAACTGCAGCTCCAGCAAAGCCACCACGCCCCTCAGCCCATTCTCGGCGCCCGCCCTCTCGCCCACCGGCTTCTGTTGCCCCAAAGGATCGCCCGTTTCCGGTTACGAGAGCAGCTCATCCACCTACAGCGACAGCGGCAGCAACTACAGCCTCAATCTCCAGCTGCATGCCGTCTACAACGACAATCTGATGTACATGCAACAGCAGCAGCAGCAGCAGCAACAGCAGCATCACCTGCAACATCAGCAGCACCTGCAGCGCTGGCTGGATCAGGAGTCCTTGGCCAC**TGCGAGGACATCATCCGTCAAC**CGACCACTGATTCTCGTGGCG**GATACTGCGCCCACAAACCTGA**CCCTGGTGGCCTAA*GAACACACACACACACACACACACACACACACACAACAAACAACACACACAATAGAAACTAAGGAAGCAGCAATCCCACCAATCGGATGTTATAATGGCAGTCACTGGGCCTGGCCTTTTAGTTCCCTAAGCCATATTTAAGCTTAATAACGTTTTTTTTAGCCCTACCACCTAAGTTTACCTTAATAAAAAATTGTATATCTAAGCGCAAACATATATCTTCCAAGCATATCAGCAAAAAAAACCCCAAACCCTAGGTATAAACCCTTCTTTTTTTTTTGGTTACACACTAAGAACAAATTAAGCTTAAGTTCAGATTTTTCTTTTTTTTTTTCTTGTATTAGCTTGTGTAAATAATTGTGTACAAAAAGTGAAACAAGGCGAAACAATTGAATTTGCTTAAGAATTTCAACGCTTCATTTCATTTGTATAACTTGTGGGAGAGTATTTTAAGAGAGAAAATGTGAAAAATGCAACTGGAAAAACTGAGAAAATTGTTAAGAGTAAAGCTTTAAAAACGAAACAAATACACACCTTTACAATACACACTTTTTTTATTTGGAAATTGAAAATTGAAAATTCTTGTGATATGACAACAACAAAACAAAATTTTAATAAAATTGTTGAAAACTGAAGCTTATTGAATATACAGCTATATTATACATAAATATATATTTACAAACACAAAAGAGAGCAAGAAATTTAACGTAATATTAAGGGTTACATTTAAAAGTTAATTATAATGATATACAATATACATATATACAATATATTTATATACAAACAAGAGGAGAACGTTTTTTTTTTTTTTTCATTGCCAAGGGAAATATTAAATGCGCCTATACATAGAGAAAGAGATATATATATCTAAATGTATGTATATATATAAAATCAGTATTTTTATATATTACATTTGACAACAACAATAATAATAATAATAATTGTGTGACCATACAACAAACAAACAACAAAATCAAAAGGAAAACGAATTGATTAAATTAACAACAGAAAACTAAAAAAACTTT*

**Specificity test results**
Overall score: Good
cDNA specificity: Good
Single primer specificity: Good
Amplifies genomic DNA: Possibly; putative amplicons within the following gene(s): [FBgn0024250](http://www.quantprime.de/minimal.php?page=primerpairproblem&primerpairid=17257381&string=FBgn0024250%5B%5Bprimerpair%2C 1%2C 1%2C 1%2C 22%2C 2569%2C 2590%2C -1%2C -1%2C 22%2C 1%2C 2612%2C 2633%2C u||||||||||||||||||||||%2C u||||||||||||||||||||||%2C 65%5D%5D&type=1&organismid=355), [Unknown](http://www.quantprime.de/minimal.php?page=primerpairproblem&primerpairid=17257381&string=Unknown%5B%5Bnottested%5D%5D&type=2&organismid=355)

***smp-30***

**Transcript identifier:** [**FBtr0083016**](http://www.quantprime.de/minimal.php?page=transcripts&subaction=geneinfo&identifier=FBtr0083016&organismid=355) - type=mRNA; loc=3R:complement(10571812..10572070,10572129..10572847,10572996..10573043); name=smp-30-RA; dbxref=FlyBase:,FlyBase_Annotation_IDs:CG7390-RA,REFSEQ:NM_079629; score=11; score_text=Strongly Supported; MD5=dc6d1b95f58824592e7e78033ab10a68; length=1026; parent=FBgn0038257; release=r5.17; species=Dmel;

**Forward primer**
Sequence: ACCGTCTTCAAGGTCAATCCAAGC (24 b)
Melting temperature: 63.7 °C
G/C content: 50 %

**Reverse primer**
Sequence: AAAGCCACCGAGGTGATTTGGG (reverse complement: CCCAAATCACCTCGGTGGCTTT) (22 b)
Melting temperature: 63.7 °C
G/C content: 54.5 %

**Amplicon**
Size: 83 b
Melting temperature: 81 °C
G/C content: 47 %
Optimal annealing temperature: 60.9 °C

**Alignment with transcript sequence**
*AGTTTGCAAACGTTAGCTGACCGAGAAGTATTCAACGGTTTCTTTAAG*ATGTCATACAAGGTTGAAGCTGTTCCCGATTCCTACGCCGCCCTGGGCGAGGGACCCCACTGGGATGTTGATCGCCAGAGTCTGTACTACGTGGACCTCGAATCCGCCGGCATTAATCGTTATGATTTCAAGCAGAACAAAGTGTACAGGGCTAAAATCGAGGGCGAGATATTTGCATCGTTCATTCTGCCGGTTGAGAACAAACCGCAGGAGTTTGCCGTAGGATGCGGTCTTCGTACGGTCATCGTCCAGTGGGATGGAGTCTCCGCAGTGGCCAAGGTCACTCGCACCCTGTTCGAGGTGCAGCCGGACCTGAAGGAAAACCGCCTTAATGATGCCAAAACCGATCCCAATGGCCGTTTTTACGGTGGCACCATGGCCGACAGTGGCGACATATTCACCCAATGGAAGGGTGAGCTCTACAGCTGGCAGGCCGGTGGACAGCCCAACGCTATCCGTAGCAAGGTGGGCATATCCAATGGCCTGGCCTGGGATGTCAAGGCCAAGAAGTTCTACTTCATCGACACCAACAACCACGAGGTATTGGCCTATGACTACAATCAGAGCACCGGCGCCGTAAGCAACCCAAAGGTCATCTTCGATCTGAGGAAGATTCGGCCCGAAGGACCATTGTTCCCTGATGGCATGACCGTAGACACCGATGGCAATATCTACGTGGCCACCTTCAATGGTGGC**ACCGTCTTCAAGGTCAATCCAAG^C**ACCGGTAAAATTCTGCTGGAGATCAAAATTCCAACCA**CCCAAATCACCTCGGTGGCTTT**TGGAGGTCCCAATTTGGATATTTTGTATGTGACAACCGCCAACAAGTTCGACCAGCCAAAACCAGCTGGTACCACCTTCCAGGTCACTGGGCTCAATGCCAAGGGTTACGCCGGCGTTAATTTGAAGATCTAG*ATTATAGACTACTTGTATAATAGCATTTAATTAAATAATCAAACATTTACTTGTATACTGAACTAA*

**Specificity test results**
Overall score: Perfect
cDNA specificity: Good
Single primer specificity: Good
Amplifies genomic DNA: No

***CG12164***

**Transcript identifier:** [**FBtr0088963**](http://www.quantprime.de/minimal.php?page=transcripts&subaction=geneinfo&identifier=FBtr0088963&organismid=355) - type=mRNA; loc=2R:join(3198913..3199008,3199437..3199616,3199681..3199956,3200012..3200855); name=CG12164-RA; dbxref=FlyBase:,FlyBase_Annotation_IDs:CG12164-RA,REFSEQ:NM_136424; score=15; score_text=Strongly Supported; MD5=29e6fd74a9e8c1b43e3226355eea2e2e; length=1396; parent=FBgn0033158; release=r5.17; species=Dmel;

**Forward primer**
Sequence: GCATGAGATCCCTAAGTCTGTTGC (24 b)
Melting temperature: 61.9 °C
G/C content: 50 %

**Reverse primer**
Sequence: TCTTCCACACCTGGACCCAATC (reverse complement: GATTGGGTCCAGGTGTGGAAGA) (22 b)
Melting temperature: 62.3 °C
G/C content: 54.5 %

**Amplicon**
Size: 135 b
Melting temperature: 87.6 °C
G/C content: 56.3 %
Optimal annealing temperature: 65 °C

**Alignment with transcript sequence**
*TAGAAGACCTCAGAGGAGGCACATATCAGCTCCTTCTGTTTTAACGCGCTGTTGGTGGTGCAAGGGATACCAA****GC*ATGAGATCCCTAAGTCTGTTG^C**TACTCAGCGCCAGCTGCGCGCTAATCATAAGCTTAGCCTACGCCCATCCCCCCGAGGGAGTGTGGAAGAAGAAGCTCACCTGGAAGGAG**GATTGGGTCCAGGTGTGGAAGA**CCGTTAAGAAGGAGGCGTGGGAAACCAAGTGGAAGAAGGTTTCGGTCCCAATATGGAAGGAAGTCAAGGTTCCCGTCTGGAAGGAGGAGAAGGTTCCCGACTGGAAGATCGTAAAGAAGCCGAAAATTGAGGAGAGAGAGGTGCCTGCCTGGAAGGAGGTTAAGGTGGCCGAATGGAAGAAGATAACGAAACCCATTTGGGTGCCCACAAAGGTCGCCGTCTGGAAGGAAATCCAGGTGCCCATTTGGAAGGAAGTGCAGGTCCCGTACTGGAAGGAAATACAAGTGCCCATTTGGAAGGAAGTACAGGTCGCAGACTGGAAGCAAATGTTTGAGCCCCAATGGGTGAAAATGGGCATTCCCGGCGAAAAGTTCTTGGGTAAGGACCACGAGGGCTGGGAGTACACCAGCCACGATCTCTGGCGCAAGAAGCTGATCTGGAAGCCCGTGTGGAAGAAGGTCTGGCGCACCGAGAAGAAGCAGGAGTGGAAGACCGAGAAGAAGCAGGAGTGGCGCACCGAGAAGAAGCAGGAATGGAAGACGGAGAAGGTGCAGGAGTGGAAGCAGGACAAGAAGCTGGAGTGGAAGGACGAGTGGATCCAGGTGTGGAAGCCCGTGAAGAAACAAATCTGGATTAAGGAGAAGCGCGAGACCTGGATCGAGGAGAAGGTTCAGATCTGGCGCACCGAGAAGCGCCAGGTGTGGGCCACCGAGAAGCGGCAGGCGTGGAAGGACGAGTGGCAGTCGGTCAACGTGCCCGTCTGGAAGGAGGTCAAGGTGCAGGAGTGGAAGAAGGTGTGGAAGCCCGTCTGGGAGAAGGTGTGGGTGCCAGTGAGCCATGGCCACGGATGGGACTAG*GTCCGCGGCAGGAGTCTCCGCTTGGCCACCAGCCAAGCGACAGCACTGATATCTGATAACTGCTCCCGCTGATCCTGCTAATCCCTTGGGGTTTGCCCCATCCTCCGCAGATCACGATCCCCACTCAAATTGTTACATAGACTTAATGTTGTAAGGCATCCCACTTCCTCCTGCATTTTGTACAAAACACCTTTCATACCTTTCGTTATCTGTTGGATTTACCCATACCCCGCCTCCTTCATCGCCGTCTCCCACAAGTGTACAAATTAGTCATGCGCGCGTTTGTCTTTAAGTTATACGAAATTCAATAAAACAACAAAAACAATAATGA*

**Specificity test results**
Overall score: Perfect
cDNA specificity: Good
Single primer specificity: Good
Amplifies genomic DNA: No

***TotA***

**Transcript iden tifier:** [**FBtr0083971**](http://www.quantprime.de/minimal.php?page=transcripts&subaction=geneinfo&identifier=FBtr0083971&organismid=355) - type=mRNA; loc=3R:join(16696758..16696821,16696908..16697427); name=TotA-RA; dbxref=FlyBase:,FlyBase_Annotation_IDs:CG31509-RA,REFSEQ:NM_080517; score=15; score_text=Strongly Supported; MD5=4d9d9f49f74e83ac34f6a554149fee7f; length=584; parent=FBgn0028396; release=r5.17; species=Dmel;

**Forward primer**
Sequence: TTCCGGTTTGCTTCAGCGTTCC (22 b)
Melting temperature: 64.5 °C
G/C content: 54.5 %

**Reverse primer**
Sequence: AGCAGCAGTGCAAAGCACATAAG (reverse complement: CTTATGTGCTTTGCACTGCTGCT) (23 b)
Melting temperature: 62.8 °C
G/C content: 47.8 %

**Amplicon**
Size: 80 b
Melting temperature: 78.4 °C
G/C content: 41.3 %
Optimal annealing temperature: 58.8 °C

**Alignment with transcript sequence**
*CA****TTCCGGTTTGCTTCAGCGTTCC****AAAAAGTCATAACCAAA*ATGAATTCTTCAACTGCT**CTTAT^GTGCTTTGCACTGCTGCT**GATTAGTCCTCTATGCATGGGCTATTCCGACGAAGATCGTGAGGCTGACAACCTTAGAATTGCTGAAATTATCAAAAACGCCCAGGACGATGACTCCAAAATCAATAGCACCCAGGAACTACTTGACATCTATAGGCGTTTATATCCCAGTTTGACCCCTGAGGAACGGGAGAGTATCGATAAGTTCGTCAACGAGCATACGGATGCCATTATAATCGATGGAGTTCCGATTCAGGGAGGTCGAAAGGCGAGGATCGTTGGAAAGATTGTATCTCCAGGTGTGAAGGGCCTTGCAACTGGATTCTTTGAAGAATTGGGTTCAAAGCTTGCTCAATTATTCACTGGTTAA*TATTGTTTTAATATTTGAGTGAAAGAAAGGAAACAACAAAGTGACATATATAAGAAAGCTAATTTAATATTGACATGTAATTATTGCCACTTAACGTTTTGTATACAACTTTTAATTAATAATTTCAAATAAAATATCAATGCACATTATTTA*

**Specificity test results**
Overall score: Perfect
cDNA specificity: Good
Single primer specificity: Good
Amplifies genomic DNA: No

***TotC***

**Transcript identifier:** [**FBtr0083972**](http://www.quantprime.de/minimal.php?page=transcripts&subaction=geneinfo&identifier=FBtr0083972&organismid=355) - type=mRNA; loc=3R:join(16698710..16698776,16698840..16699310); name=TotC-RA; dbxref=FlyBase:,FlyBase_Annotation_IDs:CG31508-RA,REFSEQ:NM_080518; score=15; score_text=Strongly Supported; MD5=fbd61144e58e250df2e35ffd3577c507; length=538; parent=FBgn0044812; release=r5.17; species=Dmel;

**Forward primer**
Sequence: TCTACTATGCCTTGCCCTGCTC (22 b)
Melting temperature: 62.2 °C
G/C content: 54.5 %

**Reverse primer**
Sequence: ATCTTCGATTCGGCGTCGTTGG (reverse complement: CCAACGACGCCGAATCGAAGAT) (22 b)
Melting temperature: 63.9 °C
G/C content: 54.5 %

**Amplicon**
Size: 126 b
Melting temperature: 83.7 °C
G/C content: 47.6 %
Optimal annealing temperature: 62.4 °C

**Alignment with transcript sequence**
*ATCAGTTTGTCTTAAACCAGTGCTCTACTAGGGTTATTAACAAA*ATGAATGCCTCCATTTC**TCTACT^ATGCCTTGCCCTGCTC**CTGATTAGTCCTTTTTGCTTGGGCTATTCTGACGAGGAAAGGGAATCTGACAGCCTTAGGGTTGCAGAAATTATTCGAACCT**CCAACGACGCCGAATCGAAGAT**CAATCGAACCCAAGAGCTGCTCGATATCTTTAGGCGCCTGACTCCCACGTTGTCCCCTGAACAAAGGGAAAAGATTGAAAGATCCATTCAGGAGCATACTGATGAAATTCTAATCGATGGAGTTCCGAGTCAGGGGGGGCGAAAGACCAAGTACGTCGGAAAAATCCTATCTCCAGTGGCACAAGGCCTTGCAGTTGGATTCTTTGAAGAACTCGGTGGCAGCCTTTCCAGGCTCTTTACTGGATAA*TCACCATTCAGGACTAAGGGATTTTGAGAAACATTAGATAAACATGTTGTAAGCTCAAGTCCAAATAAAATATTCCAGTTTGAGAAGATGATTCTTAAAATTGA*

**Specificity test results**
Overall score: Good
cDNA specificity: Good
Single primer specificity: Good
Amplifies genomic DNA: Possibly; putative amplicons within the following gene(s): [FBgn0044812](http://www.quantprime.de/minimal.php?page=primerpairproblem&primerpairid=17290107&string=FBgn0044812%5B%5Bprimerpair%2C 1%2C 1%2C 7%2C 22%2C 131%2C 146%2C -1%2C -1%2C 22%2C 1%2C 229%2C 250%2C u||||||||||||||||%2C u||||||||||||||||||||||%2C 120%5D%5D&type=1&organismid=355), [Unknown](http://www.quantprime.de/minimal.php?page=primerpairproblem&primerpairid=17290107&string=Unknown%5B%5Bnottested%5D%5D&type=2&organismid=355)
